# Comparative Proteomic Analysis of Extracellular Vesicles from Donkey Colostrum and Mature Milk

**DOI:** 10.3390/metabo15090619

**Published:** 2025-09-18

**Authors:** Simonetta Caira, Sandra Buratta, Silvia Vincenzetti, Raffaella Latella, Matteo Seccaroni, Sabrina De Pascale, Cristina Federici, Luana Lugini, Stefano Giovagnoli, Anna Maria Salzano, Carla Emiliani, Andrea Scaloni, Elisabetta Chiaradia

**Affiliations:** 1 Proteomics, Metabolomics and Mass Spectrometry Laboratory, Institute for the Animal Production System in the Mediterranean Environment, National Research Council, 80055 Portici, Italy; simonetta.caira@cnr.it (S.C.); sabrinadepascale@cnr.it (S.D.P.); annamaria.salzano@cnr.it (A.M.S.); andrea.scaloni@cnr.it (A.S.); 2 Department of Chemistry, Biology and Biotechnology, University of Perugia, 06100 Perugia, Italy; sandra.buratta@unipg.it (S.B.); raffaellalatella97@gmail.com (R.L.); carla.emiliani@unipg.it (C.E.); 3 School of Biosciences and Veterinary Medicine, University of Camerino, 62024 Matelica, Italy; silvia.vincenzetti@unicam.it; 4 Department of Veterinary Medicine, University of Perugia, 06100 Perugia, Italy; seccaronimatteo@gmail.com; 5 Preclinical Research and Clinical Trials in Hematology and Oncology Unit, Department of Oncology and Molecular Medicine, Istituto Superiore di Sanità, 00161 Rome, Italy; cristina.federici@iss.it (C.F.); luana.lugini@iss.it (L.L.); 6 Department of Pharmaceutical Sciences, University of Perugia, 06100 Perugia, Italy; stefano.giovagnoli@unipg.it

**Keywords:** donkey milk, extracellular vesicles, proteomics, colostrum, molecular cargo

## Abstract

**Background/Objectives:** Donkey milk (DM) has been considered a valuable alternative to human and bovine counterparts as well as to infant formulas. Milk extracellular vesicles (EVs) have been proposed to influence key biological processes. The purpose of this study is to provide a comprehensive characterization of the protein composition of extracellular vesicles (EVs) by extending quantitative proteomic comparisons to EVs derived from donkey colostrum (DC) and mature donkey milk (MDM). **Methods:** The EVs were isolated from DC and MDM samples, characterized, and subjected to proteomic analysis using the tandem mass tag-based quantitative approach. **Results**: In addition to typical milk proteins and EV markers, EVs from DC and MDM both contain components associated with the immune system, immune response, or promoting tissue repair, and assisting with communication between the infant and their environment. The EVs from DC were enriched in proteins associated with protein turnover, specific defense functions, and regenerative processes. **Conclusions**: Overall, the results can contribute to the broader characterization of the overall protein composition of DC and MDM and might help to predict the beneficial effects of the corresponding EVs on various mammalian cells. They may also provide valuable insights for the development of novel DM-based products for food, pharmaceutical, and biotechnological applications.

## 1. Introduction

Donkey milk (DM) has been valued since antiquity for its therapeutic and regenerative properties. Long before the advent of modern infant formulas, it was used as a substitute for maternal milk in the management of childhood disorders [1,2,3,4]. DM has low allergenic properties and a valuable nutritional composition as it contains a significant percentage of essential amino acids. Moreover, it has been recognized for its unique properties, including anti-microbial, antiviral, and antifungal effects, as well as anticancer and antioxidant properties [5,6]. Being considered one of the best substitutes for human milk, the nutritional, functional, and hypoallergenic properties of DM have been characterized by both targeted and untargeted proteomics [5,6,7]. Proteins and bioactive peptides involved in the modulation of immune defense and cell metabolism, growth, and development have been highlighted [1,5,6]. DM composition, along with post-translational protein modifications, provided a rationale to explain its higher functional similarity to human milk than to the counterpart from other species [4,8,9], and promoted its employment as a nutritional supplement to healthy and ill infants. Moreover, DM is being used in cosmetics as a functional ingredient [4,10,11]. Most of these findings were evidenced after proteomic analysis of donkey milk casein, whey, and milk fatty globule membrane (MFGM) fractions [5,6,7,9]. However, the protein content of extracellular vesicles (EVs) derived from donkey milk (DM) remains largely unexplored so far [12].

EVs are membrane-enclosed nanoparticles, which are released by almost all cell types carrying nucleic acids, proteins, and metabolites. It has been established that these nanoparticles are involved in the intercellular communication by mediating the crosstalk between releasing and recipient cells within the same organism, as well as to facilitate cross-species and inter-kingdom interactions [13,14]. EVs have been described in various biological fluids, such as serum, urine, saliva and, notably, milk, as well as from organs of plants and bacteria culture supernatants. In general, the term EVs includes diverse subsets of nanovesicles that can be classified according to their biogenesis and size: exosomes, released by multivesicular bodies (MVBs) that fuse with the plasma membrane, have a diameter ranging from 50 to 150 nm; ectosomes, or microvesicles, with a size of 100–1000 nm, generated as a result of plasma membrane’s budding process; exomeres and supermeres, which are the smallest nanoparticles with diameters less than 50 nm; and apoptotic bodies, with very large dimensions (up to 5000 nm), produced from apoptotic cells [15].

By delivering their cargo to specific recipient cells, EVs can affect cell and tissue homeostasis by exerting various effects and modulating different cell functions. In particular, milk-derived EVs have attracted the interest of researchers due to their immune-modulating, antioxidant, anti-inflammatory proprieties, and their potential anticancer activity [13,16,17,18,19,20]. It has been demonstrated that they can induce hypoxia tolerance, improve and stimulate wound healing, and regulate epithelial cell growth in different tissues [18,19,20]. Protective effects of milk-derived EVs toward the intestinal tract were reported being due to the modulation of gut microbiota, ultimately increasing mucin production and reducing inflammation [21,22,23,24]. Recently, milk-derived EVs have also been proposed as promising theranostic tools [25]. Indeed, the opportunity to engineer their cargo and their stability in the gastrointestinal tract [26,27,28,29] positions them as potential candidates for therapeutic applications as well as promising drug delivery systems. Lastly, milk-derived EVs were also used as diagnostic biomarkers for breast and metabolic disorders as their cargo reflects the physiological and pathological status of the releasing cells [25].

In this study, the proteomes of EVs isolated from donkey colostrum (DC) and mature donkey milk (MDM) were explored. The characterization of the protein cargo of these EVs might be crucial for understanding the uncovered mechanisms underlying the health-promoting effects of DC and MDM, such as their potential anti-inflammatory, antimicrobial, or immunomodulatory properties. Moreover, these findings may offer valuable insights into the broader biological and nutritional relevance of whole DM and its individual fractions. By elucidating the compositional differences between EVs from DC and MDM, this study complements previous work and enhances our understanding of protein dynamics during lactation in donkeys.

## 2. Materials and Methods

### 2.1. Donkey Milk Sampling

DC samples (20 mL) were collected from 3 donkey individuals belonging to the Amiata breed 48 h after parturition; animals were reared in a local farm (Mamma Asina, Colmurano, MC, Italy) that produces DM for human consumption according to the EU Regulation 853/2004. MDM (20 mL) was withdrawn two months after parturition from the same donkeys from which the colostrum was sampled. The animals received 1.0 kg of commercial concentrate per head daily, given once a day, along with grass hay available ad libitum and free access to fresh water. Regarding sample collection timing, it was performed in the early morning hours (between 7:00 and 9:00 a.m.), which has been identified as the most suitable time for collecting donkey milk.

MDM and DC samples were transported to the laboratory in a refrigerated container (4 °C) and processed within 2 h. After an initial clearance spin (5000× *g*, for 30 min, at 15 °C) to remove fatty layer and cellular breakdown products, the skimmed milk samples were aliquoted and stored at −80 °C until EV separation.

### 2.2. Isolation of EVs from Mature Milk and Colostrum

EVs were obtained from DC and MDM according to previous studies [30,31]. After the first centrifugation step (see above), samples were diluted 1:2 with PBS and subjected to three centrifugation steps (12,000× *g* for 1 h; 35,000× *g* for 1 h, and 70,000× *g* for 1 h) (Type 70.1 Ti rotor, Beckman Coulter, Brea, CA, USA). After each step, the supernatant was recovered and subjected to further centrifugation at a higher speed. The supernatant obtained from centrifugation at 70,000× *g* was filtered twice (with 0.45 µm and 0.22 µm filters), and then centrifuged at 100,000× *g* for 60 min. The pellets recovered by the last ultracentrifugation were resuspended in PBS and centrifuged at 100,000× *g* for 1 h (TLA 100.3 rotor, Beckman Coulter, Brea, CA, USA). The final pellets were resuspended in PBS and used for the analyses described in the following paragraphs

### 2.3. Characterization of EVs

#### 2.3.1. Scanning Electron Microscopy

Scanning Electron Microscopy (SEM) examination was carried out according to previous studies [32,33]. Briefly, the EVs isolated from DC and MDM (2 μg of total proteins) were fixed in 2.5% *w*/*v* glutaraldehyde, washed with abundant water using Vivaspin concentration devices (300,000 Da cut-off), and dried on glass coverslips at room temperature. SEM images were captured using a field emission LEO 1525 electron scanning microscope (Zeiss, Thornwood, NY, USA) equipped with a Gemini column. Prior to imaging, the samples were coated with chromium (Cr) using a high-resolution sputter Q150T ES-Quorum apparatus (24 s sputter at a current of 20 mA). Cr thickness was about 10 nm.

#### 2.3.2. Nanotracking Analysis

A NanoSight Model NS300 device (Malvern Instruments, NanoSight Ltd., Salisbury, MD, USA) was used to evaluate the number and the size distribution of the EVs obtained from DC and MDM. For each determination, five 60 s videos were recorded [34].

#### 2.3.3. Immunoblotting

Aliquots of EVs from DC and MDM (10 µg proteins) were added to sample buffer 5X [1 M Tris-HCl pH 6.8, 5% (*w*/*v*) SDS, 6% (*v*/*v*) glycerol, 0.01% (*w*/*v*) Bromophenol blue] containing 125 mM DTT. Samples were separated on 10–12% T acrylamide gels and transferred to PVDF membranes. Membranes were then saturated and incubated overnight with the following antibodies: anti-CD81, anti-CD63 and anti-Tsg101 (Santa Cruz Biotechnology, Dallas, TX, USA). HRP-linked secondary antibodies (GE Biosciences, Piscataway, NJ, USA) were probed according to the manufacturer’s instructions. Immunoblot images were captured by chemiluminescence using ECL system (GE Biosciences).

### 2.4. Proteomics

#### 2.4.1. Protein Extraction, Digestion, Labeling, and Mass Spectrometric Analysis

Proteomic experiments were carried out essentially as previously reported [35]. Aliquots of each preparation of EVs (100 µg) were sonicated in an ultrasonic bath (30 min, on ice), mixed with 5% *w*/*v* Triton X100 and centrifuged at 12,000× *g* for 5 min at 4 °C. Protein-containing water–organic solvent interface was combined with four volumes of methanol and centrifuged at 14,000× *g* for five minutes at 4 °C. Protein pellets were recovered after centrifugation at 12,000× *g* for two minutes at 4 °C and dissolved in 6 M urea, 2 M thiourea, 30 mM Tris-HCl, 10 mM dithiothreitol, and 0.1% *w*/*v* Triton X100. The separated proteins were reduced with 10 mM tris(2-carboxyethyl)phosphine for 30 min at 37 °C. They were next alkylated with 55 mM iodoacetamide for 30 min at 25 °C in the dark and were ultimately precipitated with acetone at −20 °C for the entire night. Proteins obtained after centrifugation at 8000× *g* for 10 min, at 4 °C, and solubilization in 50 mM triethylammonium bicarbonate (TEAB), were submitted to proteolysis with trypsin (enzyme/protein 1:50 *w*/*w*), at 37 °C, overnight. Resulting peptides were labeled with TMT-10 plex Reagent (Thermo-Fisher Scientific, Waltham, MA, USA). EV preparations were labeled according to the following scheme: EVs from MDM 1: TMT-127C; EVs from MDM 2: TMT-128N; EVs from MDM 3: TMT-126; EVs from DC 1: TMT-129C; EVs from DC 2: TMT-130N; EVs from DC 3: TMT-128C. The labeling reaction ongoing for 1 h was quenched by adding 8 μL of 5% *w*/*v* hydroxylamine to each tube and mixing for 15 min. Tagged peptide mixtures were combined in equal-molar ratios (1:1:1:1:1:1) for comparative studies, and they were vacuum-dried using a centrifuge evaporator (SpeedVac, Thermo Fisher Scientific, Bremen, Germany). Pooled TMT-labeled peptide mixtures were then dissolved in 0.1% *v*/*v* trifluoroacetic acid and fractionated using the PierceTM High pH Reversed-Phase Peptide Fractionation Kit (Thermo-Fisher Scientific) in accordance with the manufacturer’s instructions in order to remove unbound TMT reagents and reduce sample complexity.

Eight fractions of TMT-labeled peptides including samples from EVs from the milk and colostrum of three donkey individuals were collected, vacuum-dried, and subsequently reconstituted in 0.1% *v*/*v* formic acid for mass spectrometric analysis. These fractions were analyzed in technical triplicate using an UltiMate 3000 HPLC RSLCnano system (Dionex, Thermo Scientific, Sunnyvale, CA, USA) connected to a Q-ExactivePlus mass spectrometer through a Nanoflex ion source (Thermo Fisher Scientific). A gradient of solvent B (water/acetonitrile/formic acid 19.92/80/0.08 *v*/*v*/*v*) in solvent A (water/formic acid 99.9/0.1 *v*/*v*) was used to elute the peptides, which were loaded onto an Acclaim PepMapTM RSLC C18 column (150 mm × 75 μm ID, 2 μm particles, 100 Å pore size) (Thermo-Fisher Scientific). The flow rate was 300 nL/min. The gradient grew to 60% over 125 min and to 95% over 1 min following 20 min of column equilibration at 5% of solvent B. It remained at 95% for 8 min, and finally returned to 5% in 1 min. The mass spectrometer operated in data-dependent mode, performing a full scan (*m*/*z* range 375–1500, nominal resolution of 70,000), followed by MS/MS scans of the 10 most abundant ions. MS/MS spectra were recorded within a scan *m*/*z* range of 110–2000 with a normalized collision energy of 32%, an automatic gain control target of 100,000, a maximum ion target of 120 ms, and a resolution of 17,500. A dynamic exclusion value of 30 s was also applied.

#### 2.4.2. Bioinformatic Analysis

The Proteome Discoverer vs. 3.1 (PD3.1) software (Thermo Scientific) was used to analyze raw mass data for protein identification and relative quantification. This software allowed for searching against the UniProtKB *Equus asinus* database (61,536 protein sequences) using the Mascot algorithm v. 2.6.1 (Matrix Science, Ltd., London, UK). Peptide mass tolerance was set to ± 10 ppm and fragment mass tolerance to ± 0.05 Da. Carbamidomethylation of Cys, TMT6-plex modification of lysine, and peptide N-terminal were considered as fixed modification, whereas oxidation of Met, deamidation of Asn and Gln, and pyroglutamate formation of Gln/Glu were considered as variable modifications. The proteolytic enzyme and maximum number of missed cleavages were set to trypsin and 2, respectively. Protein candidates were deemed confidently identified if they had at least two sequenced peptides and a Mascot Score ≥ 30. Filtered results had at least a false discovery rate (FDR) of 1%. Protein abundances were estimated from the TMT reporter ion intensities in the MS/MS spectra and one-way ANOVA was applied to identify proteins with statistically significant differences in abundance among samples. A fold change of ≥1.500 or ≤0.666 and a *p*-value of ≤0.05 were considered as filtering parameters to keep only the significant altered proteins in the comparison between EVs from DC and MDM. Proteomic data were deposited to the ProteomeXchange Consortium via the PRIDE partner repository with the dataset identifier PXD067848.

Heat-map elaboration of data was carried out within the PD3.1 software, using the average linkage approach and Euclidean distance to visualize quantitative patterns among the differentially represented proteins (DRPs) in EVs from DC and MDM. Similarly, principal component analysis (PCA) of the proteomic results was performed by incorporating identified DRPs of the EVs from DC and MDM as variables, with the three donkey individuals serving as factors correlated in a two-dimensional spatial distribution.

#### 2.4.3. Functional Annotation

The TransMembrane Hidden Markov Model (TMHMM) was used to predict the transmembrane regions in all proteins identified in donkey milk-derived EVs using the TMHMM 2.0 server (https://services.healthtech.dtu.dk/services/TMHMM-2.0/) (accessed on 26 August 2025). Gene Ontology (GO) enrichment analysis of all identified proteins for biological process (BP) and cellular component (CC) was carried out by using two plugins of the Cytoscape platform (version 3.10.1), namely Cluego and Clupedia (https://cytoscape.org) (accessed on 23 June 2025) [36,37]. The functional enrichment analysis of the proteins of EVs with different abundances in DC-EVs and MDM-EVs was performed by g:profile (https://biit.cs.ut.ee/gprofiler/gost) (accessed on 8 July 2025). Both the *Equus asinus* and human databases were used to perform functional interpretation of the proteomic results in the context of potential human-relevant effects. Results from both donkey and human databases were largely consistent, supporting the relevance of predicted functions for cross-species considerations. g:Profiler (https://biit.cs.ut.ee/gprofiler/convert) (accessed on 22 June 2025) was used to convert gene ID lists as needed.

## 3. Results

### 3.1. Characterization of EVs from DC and MDM

**Figure 1:**
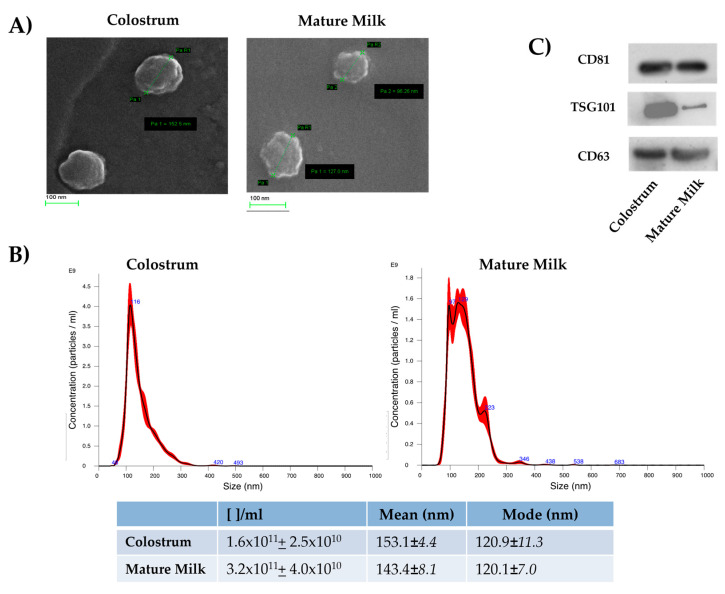
Morphological characterization and size distribution analysis of EVs isolated from DC and MDM. (**A**) Representative SEM images. (**B**) Representative quantification of EVs. Number of particles/mL ([ ]/mL), mean (nm), and mode (nm) are reported in table. Data are expressed as mean ± SD (n = 4). (**C**) EVs probed by Western blotting for well-known vesicle markers.

EVs isolated from DC and MDM have been biophysically characterized through SEM and NTA. SEM images showed that both DC-EVs and MDM-EVs presented rounded morphology (Figure 1A). Both types of EVs exhibited a size range between 100 and 300 nm, with a mean size of 153 ± 4.4 and 143.4 + 8.2 nm for DC-EVs and MDM-EVs, respectively (Figure 1B). The numbers of vesicles per ml of starting material were 7.3 × 10^10^ (+9.3 × 10^9^) and 3.7 × 10^10^ (+5.9 × 10^9^) for EVs from DC and MDM, respectively (Figure 1B), whereas the EV yields, expressed as the ratio of EV number to protein, were consistent with the range 10^10^ particles/µg of proteins for DC-EVs and 10^9^ particles/µg of proteins for MDM-EVs. Western blotting showed that DC-EVs and MDM-EVs were both positive to vesicular markers CD63, CD81, and Tsg101 (Figure lC).

These results demonstrated that the EVs from DC and MDM presented biophysical (morphology, size distribution, and number of particles/µg of proteins) and biochemical features (presence of EV markers) similar to those of EVs isolated by ultracentrifugation from the milk and colostrum of other species [38,39,40].

### 3.2. Proteomic Analysis of EVs from DC and MDM

**Figure 2:**
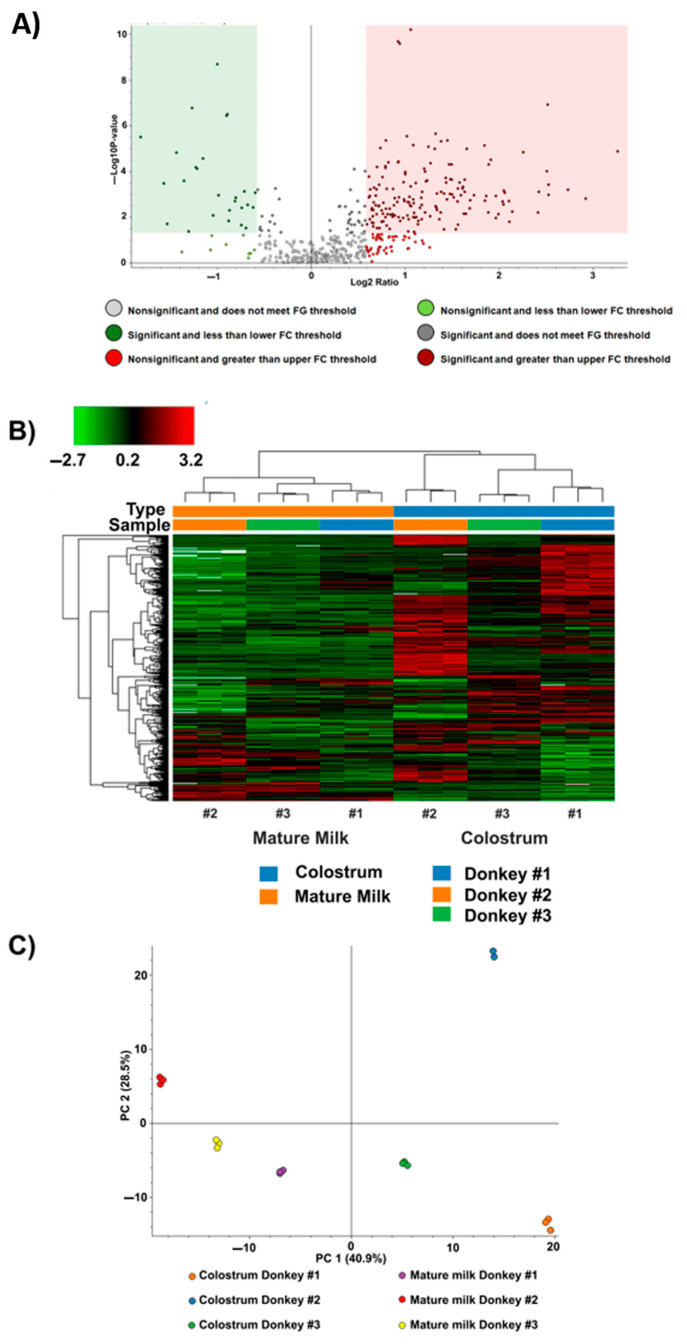
An overview of the proteomic results. (**A**) A volcano plot depicting the differentially abundant proteins with a 0.666 ≤ fold-change ≥ 1.500 and a *p*-value ≤ 0.05. Within the areas of significance, 164 and 26 proteins were more abundantly represented in DC-EVs (pink zone) and MDM-EVs (green zone), respectively. (**B**) A heat-map showing the variable abundance of DRPs in EVs isolated from MDM and DC of the three donkey individuals. Each column corresponds to a technical replicate of the three individuals, whereas each row represents a single protein. Increasing brightness towards red indicates higher protein responses (measured as summed peak areas), whereas green indicates lower protein responses. The dendrograms from the unsupervised hierarchical cluster analysis of the columns and the rows (using the Euclidean distance function and average linkage method) illustrate the similarity of the donkey individuals and proteins. (**C**) Principal component analysis (PCA) score plot of DRPs in EVs from MDM and DC of the three donkey individuals. The separation of the protein level data (normalized abundances) was evident. Each point in the chart describes a technical replicate. PCA explained about 56.1% of the variability of the different samples.

The EVs isolated from DC and MDM were characterized through to gel-free quantitative tandem mass tag (TMT) approach, according to what was reported in the experimental section. A total of 505 proteins was identified in both DC-EV and MDM-EV typologies, among which some corresponded to the same different accession numbers that were associated with products of the same gene. Quantitative comparison between EVs of DC and MDM revealed a total of 190 DRPs with a fold change ≥1.500 or ≤0.666 and a *p*-value ≤ 0.05. Among the latter components, 164 were more abundantly present in DC-EVs, whereas 26 proteins were over-represented in MDM-EVs (Figure 2A), with the remaining 315 not showing significant quantitative changes (Supplementary Materials Table S1). As expected, heat-map representation revealed that EVs from DC generally had higher protein expression levels than MDM counterparts. Certain protein clusters were specifically over-represented in colostrum vesicles, pointing to their functional relevance in early lactation (Figure 2B).

PCA of the normalized protein abundances from the three match pairs of DC-EVs and MDM-EVs and samples provided an overview of the measured proteomic variations (Figure 2C). The PCA clearly separated EVs from DC and MDM for all three donkeys. Within each matrix, the proteomes of two animals clustered more closely, whereas the third displayed distinct features.

TMHMM analysis of DM-EVs showed that 98 out of the 505 identified proteins are predicted to contain transmembrane domains (Supplementary Materials Table S2). Specifically, 31 transmembrane proteins were found among the 164 proteins more abundant in DC-EVs, 3 among the 26 proteins less abundant in MDM-EVs, and 64 among the 315 proteins with similar levels in EVs from both DM and MDM. This suggests that the majority of surface-exposed proteins are maintained high across lactation as well as the capacity of EVs to be taken up by recipient cells.

### 3.3. Comprensive Description of Protein Cargo of EVs from DC and MDM

**Figure 3:**
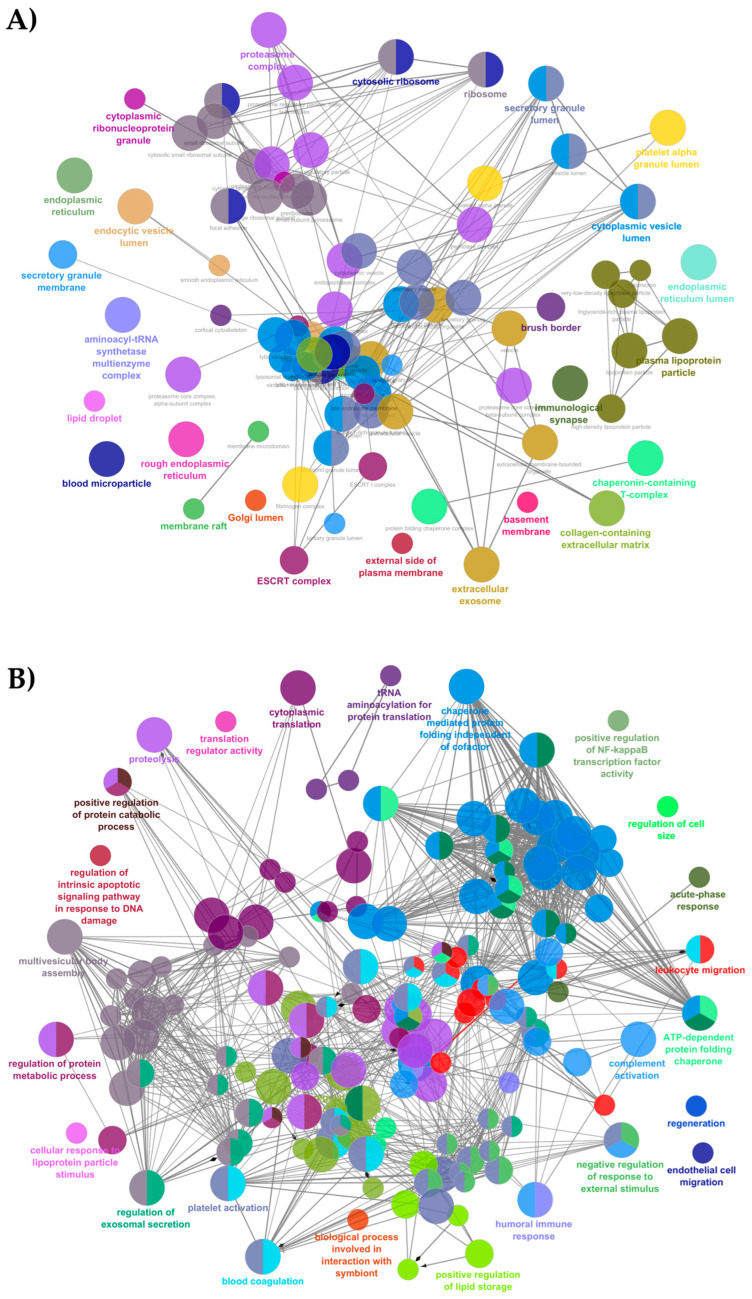
Gene Ontology analysis of all proteins identified in EVs from DC and MDM as performed using ClueGO and CluePedia plugins in Cytoscape. (**A**) Cellular Component (CC) annotations; (**B**) Biological Process (BP) annotations.

Typical mature milk proteins such as αs1-, αs2-, β- and κ-casein, β-lactoglobulin, α-lactalbumin (α-LA), lactadherin, butyrophilin, lactoferrin, lysozyme C (Lyz), and xanthine dehydrogenase were detected in EVs from both DC and MDM. Most of them were already identified in EVs from mature milk and colostrum preparations of other animals [12,41,42]. Their detection was not surprising because some of them are constitutive components of larger lipophilic particles, i.e., fat globules, or were already found absorbed on them [43]. Specific EV protein markers, including those previously detected by Western blotting, were also identified. In particular, CD81, CD63, CD9 CD82, CD151, tetraspanin-6, tetraspanin-14, and tetraspanin-2 were detected in vesicles from both DC and MDM, together with members of the endosomal sorting complex required for transport (ESCRT) including tumor susceptibility gene 101 protein (Tsg101), vacuolar protein sorting-associated protein 28 (VPS28), multivesicular body subunit 12A (MVB12A), charged multivesicular body proteins 2A, 2B and 5 (CHMP2A, CHMP2B and CHMP5), vesicle-fusing ATPase (VPS4A), and programmed cell death 6 interacting protein (PDCD6IP/Alix). Coherently, a comprehensive and deep GO analysis for CC terms, which was performed using Cytoscape with *p* < 0.05, and excluding entries with a number of associated genes lower than 5, evidenced term categories related to EVs and EV trafficking (Figure 3A). In parallel, “regulation of exosomal secretion” and “multivesicular body assembly” were highlighted as protein categories related to EV trafficking when BP terms were considered (Figure 3B). Since all the proteins were commonly identified in EVs from DC and MDM, no differences were observed in the graphs of the two sample typologies.

As expected, multiple proteins were included in BP and CC terms linked to the promotion of the defense response and immune tolerance, and the protection of the neonatal gut from over-reactive immune challenges (Figure 3B). Other BP terms related to complement and blood coagulation were also evidenced (Figure 3B).

Enriched CC and BP categories related to protein turnover included “proteasome complex” and “ribosome” (Figure 3A), as well as “cytoplasmatic translation” and “proteolysis” (Figure 3B), respectively. Finally, enriched BP categories associated with cellular and tissue homeostasis were also observed like “endothelial cell migration” and “regeneration” (Figure 3B).

Overall, the results demonstrated that EVs from DC and MDM contain proteins that serve as critical mediators of immune regulation, metabolic support, and cellular and tissue homeostasis. The identified proteins in EVs might play pivotal roles in optimizing conditions for neonatal development while providing protection against environmental challenges.

### 3.4. Differential Protein Cargo of EVs from DC and MDM

**Figure 4:**
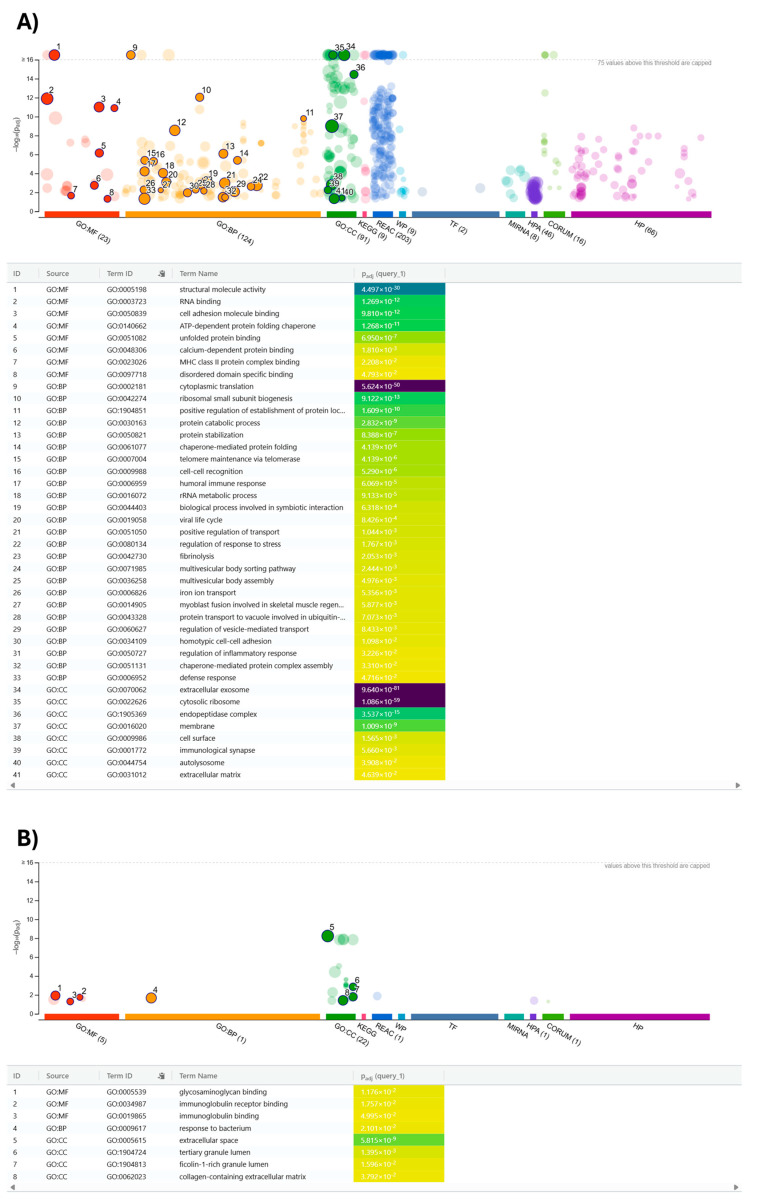
Functional profiling of proteins ascertained in EVs from DC and MDM after bioinformatic analysis with g:profile (https://biit.cs.ut.ee/gprofiler/gost, accessed on 8 July 2025) to highlight driver terms in GO. (**A**) Proteins with augmented representation in EVs from DC; (**B**) proteins with augmented representation in EVs from MDM.

With the aim of describing the main functional differences associated with the composition changes in EVs from DC and MDM, a dedicated GO analysis for significant Biological Processes (BPs), Molecular Functions (MFs), and Cellular Components (CCs) was performed, focusing on 164 components more abundantly present in DC-EVs, 26 proteins over-represented in MDM-EVs, and the remaining 315 molecular species not showing significant quantitative changes in MDM-EVs and DC-EVs; the corresponding graphical representations are depicted in Figure 4A and B, and Supplementary Material Figure S1, respectively. In particular, the proteins more abundantly represented in EVs from DC showed a strong association with immune-related functions, with enrichment in terms like “MHC class II protein complex” and “humoral immune response, defense response, and immunological synapse” (Figure 4A). A similar finding was observed in the case of augmented proteins involved in cellular adhesion and communication. Altogether, these findings suggest that a general, constant high representation of components associated with immune and cell adhesion/binding functions was observed in EVs from colostrum and mature milk, although the concentration of specific functional effectors passing from DC to MDM varied. On the other hand, the EVs from DC specifically showed augmented levels of proteins involved in protein synthesis, degradation, and quality control mechanisms (Figure 4A). Proteins associated with EV biology were also significantly over-represented. Finally, augmented levels in EVs from DC for proteins linked to some regulatory roles were observed. This suggests a potential role of the EVs from DC in modulating intracellular trafficking and facilitating adaptation to stress in recipient cells.

The different colors correspond to distinct functional annotation categories automatically assigned by g:Profiler: red = GO:MF (Molecular Function), orange = GO:BP (Biological Process), green = GO:CC (Cellular Component), blue = KEGG/Reactome pathways, etc. The unselected terms are transparent. The color gradient in the 'p.adj' column of the table reflects the statistical significance of enrichment (from less significant in green/yellow to highly significant in purple). The proteins more abundantly represented in EVs from MDM showed a significant association with immune-related functions (Figure 4B). Indeed, GO terms related to immunoglobulin binding and response to bacterial challenge were highlighted. The observed term “tertiary and ficolin-1-rich granule lumen” also refers to granule-derived immune proteins that clearly have protective and defensive functions. Moreover, over-represented ECM-related proteins, namely transforming growth factor-beta-induced protein ig-h3 (TGFBI), extracellular matrix protein 2 (ECM2), collagen type XV alpha 1 chain (COL15A1), and cadherin-1 (CDH1), were suggestive of a specific role of EVs from MDM in promoting tissue remodeling, cell adhesion, and potential signaling interactions with host tissues (e.g., gut epithelium) (Figure 4B). Finally, a role in supporting lipid metabolism and energy balance for EVs from MDM was suggested by the observed augmented concentration of fatty acid binding protein 3 (FABP3), lipoprotein lipase (LPL), phosphoglucomutase-1 (PGM1), and secreted phosphoprotein 1.

Regarding proteins having comparable abundance in EVs from DC and MDM, the number of components associated with “extracellular vesicles” and related terms including “vesicle-mediated transport”, “late endosome to lysosome transport”, “multivesicular body assembly”, and “ESCRT III complex disassembly” is worth mentioning, as it underscores their common role in allowing vesicular trafficking and functioning of the endosomal machinery involved in EV biogenesis and release, notwithstanding the case of DC or MDM (Supplementary Material Figure S1). A similar consideration can be made for proteins implicated in lipid metabolism and transport, as suggested by the enriched terms “lipid transfer activity”, “regulation of lipid localization”, “phospholipid efflux”, “triglyceride metabolic process”, and “lipid droplet” in components showing comparable abundance in EVs from DC and MDM.

The observation of stable protein cargo in EVs from DC and MDM also included components linked to oxidative stress defense and detoxification, as evidenced by the enrichment in “antioxidant activity”, “thioredoxin peroxidase activity”, “molecular sequestering activity”, “reactive oxygen species metabolic process”, and “cellular detoxification” terms, indicating a potent redox buffering capacity of EVs, notwithstanding the case of mature milk or colostrum (Supplementary Materials Figure S1). A similar consideration can be made for constant components in EVs from DC and MDM implicated in the modulation of cell architecture and dynamics, as shown by the enriched terms “cell adhesion molecule binding”, “calcium-dependent protein binding”, “actin cytoskeleton organization”, “cell junction organization”, “establishment or maintenance of cell polarity”, “focal adhesion”, and “actin cytoskeleton”.

Constant immune and interspecies signaling functions in EVs from DC and MDM were suggested by the enrichment in “lipopolysaccharide-mediated signaling pathway”, “cellular response to diacyl bacterial lipopeptide”, and “biological process involved in interspecies interaction” terms (Supplementary Material Figure S1), highlighting the common immunomodulatory potential of EVs in mucosal environments independently whether present in DC and MDM. A similar condition was observed for other terms associated with signaling functions, such as “GTPase activity”, “Cdc42 protein signal transduction,” and “molecular function regulator activity”. Finally, a common quantitative representation in EVs from DC and MDM was observed for proteins related to tissue remodeling and regeneration, with enriched terms including “cell population proliferation”, “modification of postsynaptic structure”, “protein metabolic process”, “structural molecule activity” and “wound healing” (Supplementary Materials Figure S1), implying identical trophic and regenerative roles of both EVs during postnatal development and mucosal repair, notwithstanding the case of mature milk or colostrum.

## 4. Discussion

In the present study, a comparative investigation was accomplished on EVs from DC and MDM. Despite the limited number of examples in this context, dedicated studies describing the proteome of EVs from sheep, bovine, human, buffalo, and porcine milk demonstrated that these vesicles contain a significant representation of various protein components [12,42,44,45]. The protein cargo of milk-derived EVs seemed to change according to lactation time and to be enriched in different proteins, similarly to what was observed in other species [46,47,48]. These quantitative changes were also associated with the physio-pathological condition of the corresponding animals [49,50]. Growing evidence suggests that milk-derived EVs may be considered a new functional food due to their health-promoting effects, as demonstrated by in vitro and in vivo studies [13,20]. To date, the careful description of the protein cargo of milk-derived EVs remains an open area of investigation in milk proteomics, as the available data are limited compared to those for MFG, whey, or casein fractions.

In this study, biophysical analyses demonstrated that EVs from DC and MDM have a similar morphology and size distribution. The measured ratio of EV number to total proteins, which is a good indicator of purity, was around 10^9^ particles/μg of proteins, in agreement with similar values ascertained for ultracentrifuged EVs from bovine milk [38].

Gene Ontology (GO) analysis of protein cargo of EVs from DC and MDM confirmed a significant enrichment in “extracellular vesicles” functions. Some of these proteins were already recognized as components of the ESCRT complex, which is crucial for the biogenesis and release of exosomes [51]. The occurrence of these proteins clearly suggests that the vesicle samples here described were enriched in specific EV subtypes originating from the endosomal compartment. Moreover, EVs from DC and MDM also contained other EV markers, including CDs and tetraspanins, which are known to assist vesicle formation and stability. A high number of transmembrane proteins (98 out of 505 identified) were identified in DM-EVs, with most of them showing similar levels in EVs from both DC and MDM.

EVs from DC and MDM also contained classical milk proteins (e.g., αs1-, αs2-, β- and κ-caseins, β-lactoglobulin, lactostansferrin, lactadherin, xanthine dehydrogenase, and bu-tyrophilin), which were already identified in the EVs from colostrum and mature milk preparations of other animals [12]. Their detection was not surprising because some of them are constitutive components of larger lipophilic particles, i.e., fat globules, or were already found absorbed on them [43]. The unexpected over-representation of αs1- and αs2-caseins in EVs from DC raised the recurring question on whether the caseins detected in milk EV preparations belong to genuine cargo proteins or are contaminants co-isolated with these vesicles. If passive carry-over was the sole explanation of this contamination, the EVs’ proteome should have mirrored the native casein-to-whey ratio of each fluid (about 1:1 in DC and about 1.2–1.3 in MDM) [52]. Instead, EVs from DC were markedly enriched in αs1- and αs2-caseins, whereas β-casein remained quantitatively unchanged. Although it has been recognized that MFGs and casein micelles may serve as sources of contamination, this study opted to avoid methods for micelle dispersion, such as the use of calcium chelators (e.g., EDTA) or enzymes, as they can alter the membrane proteins of EVs [20]. Nevertheless, a selective encapsulation mechanism that packages specific casein isoforms into neonatal colostrum vesicles cannot be excluded at present.

Other important proteins found in EVs from DC and MDM included α-LA and Lyz. These proteins contribute significantly to the nutritional and functional value of DM within the food chain [4]. α-LA contributes significantly to the hypoallergenic properties of DM as well as to its nutritional and immunological characteristics. Lyz significantly influences the overall immunological profile of DM, offering immune protection to the newborn like that elicited in human fluid. Related immuno-stimulating properties of the EVs from DC and MDM could be associated with mannose-binding protein C (MBL2) and MBL-associated serine protease 1 (MASP1), which both play a central role in the innate immune system thanks to their anti-bacterial and anti-inflammatory activities. MBLs participate as opsonins in the innate immune system of mammals, inducing the phagocytosis of targets (e.g., bacteria) by binding them and then also linking phagocytic receptors on phagocytes [53]. Thus, opsonins promote the interaction between the phagocytic receptor and its targets, inducing their engulfment and phagocytosis. As expected, the role of these proteins in milk has been linked to the protection of newborns from pathogens. Moreover, we have detected tyrosine-protein kinase (Lyn) in EVs from DC and MDM; this molecule is a non-receptor tyrosine kinase that regulates the development and function of various immune cells [54]. It was already described as a key modulator of the mucosal immune system and of the pathophysiological state in various models of intestinal disease [55]. A further contribution to the innate immune response can be claimed for Toll-like receptor 2 and S100A12 (also named as calgranulin), which we observed as proteins equally abundant in EVs from both MDM and DC, or over-represented in EVs from DC, respectively. The latter compound is a well-known antimicrobial peptide secreted by various cell types, including epithelial cells, which is involved in the regulation of inflammatory processes and immune response [56].

The protein cargo of EVs from MDM and DC was also shown to include other molecules playing a role in the defense against pathogens. This is the case of complement C3, C5, and C9, with the former protein showing augmented abundance in EVs from DC. Higher levels of complement proteins were already reported in DM, with respect to human and bovine milk [9]. Other proteins involved in the modulation of complement activation were observed to augment the levels in EVs from DC, including plasmin (PLG) and vitronectin (VTN), which contribute to the regulation of coagulation and hemostasis in the newborn [46] and were already associated with some toxicity of bovine milk EVs [57]. Coherently, we found higher content of fibrinogen alpha, beta, and gamma chains (FGA, FGB, and FGG) in EVs from DC, whereas fibulin 1 (FBLN1) and antithrombin III (SERPIN1) showed similar amounts in EVs from DC and MDM. All these proteins could be involved in the modulation of the neonatal hemostatic system, which is crucial for the newborn [58]. The results reported above on the selective enrichment of specific proteins involved in fibrin clot formation and complete activation in EVs from DC are similar to previous studies on EVs from human and bovine colostrum [48]. Anyway, the role of EVs in the regulation of coagulation needs additional dedicated studies [59,60].

EVs from DC seem to highly contribute to the iron supply to the newborn, as evidenced by the 7.6-fold ferritin content in these vesicles with respect to the counterpart from MDM. This finding aligns with the proposed role of the mammary gland as a source of iron for the newborn. Although ferritin is primarily known as an intracellular iron storage protein, it appears to also be secreted into the extracellular space via EVs [61] through a CD63-mediated mechanism [62]. Higher levels of ferritin were also described in EVs from sows’ colostrum compared to mature milk [63]. Other protein related to iron metabolism, including lactotransferrin and transferrin, showed similar abundance in EVs from DC and MDM.

Many ribosomal proteins were identified in EVs from DC and MDM, among which various ones showed augmented levels in the DC-EVs, which is in agreement with comparative proteomic determinations on exosomes from human and bovine milk and colostrum [47]. Although these proteins are well-known for their roles in translation, growing evidences suggest other functions in the extracellular space [64,65]. It has been proposed that extracellular ribosomal proteins, carried by exosomes, can profoundly influence the phenotype of recipient cells by interacting at the molecular level with signaling pathways such as those related to NF-κB and other mediators, thereby contributing to several pathological processes including tumorigenesis and drug resistance [59]. They have been deemed as typical components of exosomes and have also been described in EVs from the milk of other species [13,66]. The high quantitative representation of ribosomal proteins might be associated with the high polypeptide biosynthetic rate of the lactating mammary gland, thus reflecting the physiological status of the latter [67]. Additional proteins related to protein turnover, including translation factors, proteasome components, and aminoacyl tRNA ligases, were also detected, with a higher abundance observed in EVs from DC. All these proteins can be related to the role of milk vesicles in the promotion of neonatal growth [13,48] and to their rRNA cargo [68]. The high content in proteins involved in protein turnover, together with the different types of collagens and growth factors, could also be related to the regenerative properties of DM [11] and of the corresponding EVs [69].

Coherently with what was observed in porcine milk EVs [46], proteins related to lipid intake and utilization, including FABP3 and LPL, were found to be enriched in EVs from MDM. Moreover, apolipoproteins, including APO-AI, APO-AII, APO-AIV, APO-B, APO-CII, APO-E, and APO-D, were identified to have similar levels in EVs from DC and MDM, even though some of these apolipoproteins were reported to show augmented concentration in the colostrum of various animal species [70,71,72], including donkeys [5]. Recently, it has been proposed that, in addition to their role in lipid metabolism, apolipoproteins can have a role in immune defense, intestinal protection, and modulation of tissue growth [73,74].

## 5. Conclusions

In this study, the protein cargo of EVs from DC and MDM was characterized and quantitatively compared. In general terms, EVs from DC appeared richer in proteins involved in protein turnover, specific defense functions, cell adhesion/communication, stress response, and regeneration. The EVs from both DC and MDM showed similar levels of components playing a pivotal role in the immune system and in the modulation of immune response. Both EVs also contained protein involved in oxidative stress response, cytoskeletal remodeling, proper organ development, and wound healing. These features reinforce the idea that milk EVs are functional particles that contribute to the nutritional and immunological shaping of the neonatal environment, especially at the mucosal interface, like in the gut.

The relatively small number of biological replicates could represent a limitation of this study, mainly due to ethical and logistical constraints associated with sample collection from donkeys. Nonetheless, the findings of this investigation offer valuable preliminary insights and provide a foundation for future studies with larger cohorts. They provide a valuable contribution to the understanding of properties of DM, as many of the proteins here identified were never described before, adding new information about the species-specific characteristics of donkey products. Furthermore, the characterization of the protein cargo of EVs from DC and MDM can be crucial in developing novel DM-based products for food, cosmeceutical, pharmaceutical, and biotechnological applications. DM, often considered one of the earliest nutraceutical products and natural cosmetics, holds promise for a wide range of innovative purposes, in which EVs can play a key role. Future work might include the progressive validation of this preliminary study, beginning with in vitro assays, followed by in vivo animal studies, and ultimately clinical investigations in humans, to fully elucidate the biological relevance of the information here reported. While numerous studies on EVs from the milk of other animal species have highlighted their theranostic potential, concerns about potential toxicity and/or adverse effects remain the subject of ongoing debates.

## Data Availability

All proteomic data supporting this study were deposited to the ProteomeXchange Consortium via PRIDE under accession PXD067848.

## Supplementary Materials

The following supporting information can be downloaded at: https://www.mdpi.com/article/10.3390/metabo15090619/s1. Table S1: List of identified and differentially abundant protein species detected in the from DC and MDM; Table S2: TransMembrane Hidden Markov Model (TMHMM) of protein identified in DM; Figure S1: Functional profiling of proteins with unchanged representation in EVs from DC and MDM.

## Abbreviations

The following abbreviations are used in this manuscript:

DM	Donkey Milk
EVs	Extracellular Vesicles
MDM	Mature Donkey Milk
DC	Donkey Colostrum
MFGM	Milk Fatty Globule Membrane
SEM	Scanning Electron Microscopy
TMT	Tandem Mass Tags
NTA	Nanotracking Analysis
FDR	False Discovery Rate
PCA	Principal Component Analysis
DRPs	Differentially Represented Proteins
TMHMM	TransMembrane Hidden Markov Model
GO	Gene Ontology
BP	Biological Process
CC	Cellular Component
MFs	Molecular Functions
α-LA	Alpha-Lactalbumin
FABP3	Fatty Acid Binding Protein 3
MBL	Mannose-Binding Protein
LPL	Lipoprotein Lipase
Lyz	Lysozyme C
