# Supplementary material for: Comparative Proteomic Analysis of Extracellular Vesicles from Donkey Colostrum and Mature Milk
Source: Metabolites. 2025 Sep 18;15(9):619. doi: 10.3390/metabo15090619 (PMC12472028; doi:10.3390/metabo15090619)
Supplement: Supplementary file 1 [file metabolites-15-00619-s001.zip › Figure S1.pdf]

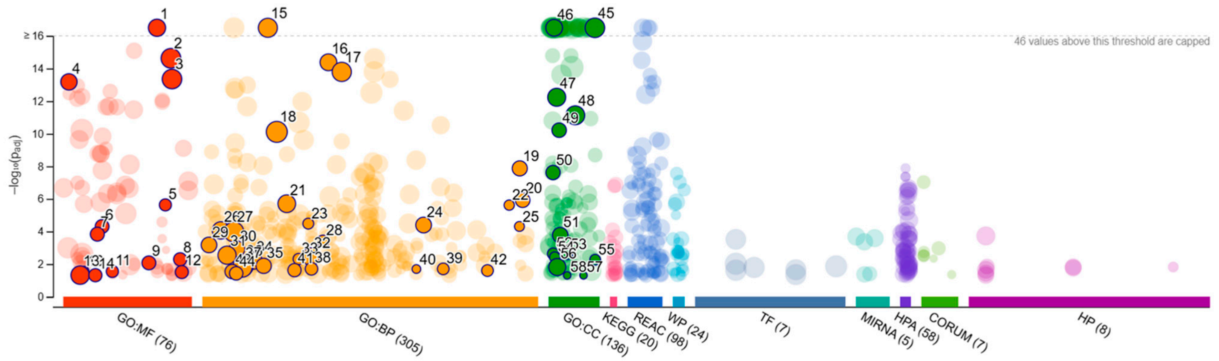


| ID | Source | Term ID | Term Name | $p_{adj}$ (query_1) |
|---|---|---|---|---|
| 1 | GO:MF | GO:0050839 | cell adhesion molecule binding | $2.285\times10^{-20}$ |
| 2 | GO:MF | GO:0097367 | carbohydrate derivative binding | $2.357\times10^{-15}$ |
| 3 | GO:MF | GO:0098772 | molecular function regulator activity | $4.430\times10^{-14}$ |
| 4 | GO:MF | GO:0003924 | GTPase activity | $6.605\times10^{-14}$ |
| 5 | GO:MF | GO:0070325 | lipoprotein particle receptor binding | $2.338\times10^{-6}$ |
| 6 | GO:MF | GO:0017022 | myosin binding | $4.909\times10^{-5}$ |
| 7 | GO:MF | GO:0016209 | antioxidant activity | $1.462\times10^{-4}$ |
| 8 | GO:MF | GO:0120013 | lipid transfer activity | $5.114\times10^{-3}$ |
| 9 | GO:MF | GO:0048306 | calcium-dependent protein binding | $8.456\times10^{-3}$ |
| 10 | GO:MF | GO:0008379 | thioredoxin peroxidase activity | $2.618\times10^{-2}$ |
| 11 | GO:MF | GO:0031210 | phosphatidylcholine binding | $2.819\times10^{-2}$ |
| 12 | GO:MF | GO:0140313 | molecular sequestering activity | $3.085\times10^{-2}$ |
| 13 | GO:MF | GO:0005198 | structural molecule activity | $4.787\times10^{-2}$ |
| 14 | GO:MF | GO:0015485 | cholesterol binding | $4.831\times10^{-2}$ |
| 15 | GO:BP | GO:0016192 | vesicle-mediated transport | $9.710\times10^{-20}$ |
| 16 | GO:BP | GO:0042060 | wound healing | $4.152\times10^{-15}$ |
| 17 | GO:BP | GO:0044419 | biological process involved in interspecies interact... | $1.605\times10^{-14}$ |
| 18 | GO:BP | GO:0019538 | protein metabolic process | $7.712\times10^{-11}$ |
| 19 | GO:BP | GO:1905952 | regulation of lipid localization | $1.342\times10^{-8}$ |
| 20 | GO:BP | GO:1990748 | cellular detoxification | $1.209\times10^{-6}$ |
| 21 | GO:BP | GO:0030036 | actin cytoskeleton organization | $1.965\times10^{-6}$ |
| 22 | GO:BP | GO:1904903 | ESCRT III complex disassembly | $2.415\times10^{-6}$ |
| 23 | GO:BP | GO:0033700 | phospholipid efflux | $3.299\times10^{-5}$ |
| 24 | GO:BP | GO:0072593 | reactive oxygen species metabolic process | $4.028\times10^{-5}$ |
| 25 | GO:BP | GO:1905920 | positive regulation of CoA-transferase activity | $4.909\times10^{-5}$ |
| 26 | GO:BP | GO:0003013 | circulatory system process | $8.586\times10^{-5}$ |
| 27 | GO:BP | GO:0006796 | phosphate-containing compound metabolic proce... | $1.066\times10^{-4}$ |
| 28 | GO:BP | GO:0036258 | multivesicular body assembly | $4.012\times10^{-4}$ |
| 29 | GO:BP | GO:0001906 | cell killing | $6.707\times10^{-4}$ |
| 30 | GO:BP | GO:0007163 | establishment or maintenance of cell polarity | $1.187\times10^{-3}$ |
| 31 | GO:BP | GO:0006082 | organic acid metabolic process | $2.858\times10^{-3}$ |
| 32 | GO:BP | GO:0034330 | cell junction organization | $3.504\times10^{-3}$ |
| 33 | GO:BP | GO:0032488 | Cdc42 protein signal transduction | $4.909\times10^{-3}$ |
| 34 | GO:BP | GO:0010256 | endomembrane system organization | $6.080\times10^{-3}$ |
| 35 | GO:BP | GO:0015718 | monocarboxylic acid transport | $1.281\times10^{-2}$ |
| 36 | GO:BP | GO:0009056 | catabolic process | $1.334\times10^{-2}$ |
| 37 | GO:BP | GO:0008283 | cell population proliferation | $1.611\times10^{-2}$ |
| 38 | GO:BP | GO:0034389 | lipid droplet organization | $1.967\times10^{-2}$ |
| 39 | GO:BP | GO:0099010 | modification of postsynaptic structure | $1.976\times10^{-2}$ |
| 40 | GO:BP | GO:0071726 | cellular response to diacyl bacterial lipopeptide | $2.014\times10^{-2}$ |
| 41 | GO:BP | GO:0031663 | lipopolysaccharide-mediated signaling pathway | $2.362\times10^{-2}$ |
| 42 | GO:BP | GO:1902774 | late endosome to lysosome transport | $2.498\times10^{-2}$ |
| 43 | GO:BP | GO:0006641 | triglyceride metabolic process | $2.883\times10^{-2}$ |
| 44 | GO:BP | GO:0007080 | mitotic metaphase chromosome alignment | $3.722\times10^{-2}$ |
| 45 | GO:CC | GO:1903561 | extracellular vesicle | $1.385\times10^{-145}$ |
| 46 | GO:CC | GO:0005925 | focal adhesion | $9.156\times10^{-32}$ |
| 47 | GO:CC | GO:0009986 | cell surface | $5.802\times10^{-13}$ |
| 48 | GO:CC | GO:0045202 | synapse | $7.407\times10^{-12}$ |
| 49 | GO:CC | GO:0022626 | cytosolic ribosome | $6.077\times10^{-11}$ |
| 50 | GO:CC | GO:0005811 | lipid droplet | $2.413\times10^{-8}$ |
| 51 | GO:CC | GO:0030496 | midbody | $1.680\times10^{-4}$ |
| 52 | GO:CC | GO:0005828 | kinetochore microtubule | $2.337\times10^{-3}$ |
| 53 | GO:CC | GO:0034663 | endoplasmic reticulum chaperone complex | $2.544\times10^{-3}$ |
| 54 | GO:CC | GO:0008540 | proteasome regulatory particle, base subcomplex | $3.777\times10^{-3}$ |
| 55 | GO:CC | GO:1904930 | amphisome membrane | $5.400\times10^{-3}$ |
| 56 | GO:CC | GO:0015629 | actin cytoskeleton | $1.521\times10^{-2}$ |
| 57 | GO:CC | GO:0072563 | endothelial microparticle | $4.971\times10^{-2}$ |
| 58 | GO:CC | GO:0034515 | proteasome storage granule | $4.971\times10^{-2}$ |

**Figure S1.** Functional profiling of the proteins with unchanged representation in EVs from donkey colostrum (DC) and mature donkey milk (MDM), after bioinformatic analysis with g:profile (https://biit.cs.ut.ee/gprofiler/gost) to highlight driver terms in GO. The different colors correspond

to distinct functional annotation categories automatically assigned by g:Profiler: red = GO:MF (Molecular Function), orange = GO:BP (Biological Process), green = GO:CC (Cellular Component), blue = KEGG/Reactome pathways, etc. The unselected terms are transparent. The color gradient in the 'p.adj' column of the table reflects the statistical significance of enrichment (from less significant in green/yellow to highly significant in purple).
